# Understanding Health Empowerment From the Perspective of Information Processing: Questionnaire Study

**DOI:** 10.2196/27178

**Published:** 2022-01-11

**Authors:** Fei Jiang, Yongmei Liu, Junhua Hu, Xiaohong Chen

**Affiliations:** 1 Business School of Central South University Changsha China; 2 Hunan University of Technology and Business Changsha China

**Keywords:** online health information, perceived argument quality, perceived source credibility, health literacy, health empowerment, information seeking

## Abstract

**Background:**

Massive, easily accessible online health information empowers users to cope with health problems better. Most patients search for relevant online health information before seeing a doctor to alleviate information asymmetry. However, the mechanism of how online health information affects health empowerment is still unclear.

**Objective:**

To study how online health information processing affects health empowerment.

**Methods:**

We conducted a cross-sectional questionnaire study that included 343 samples from participants who had searched online health information before the consultation. Respondents' perceptions of online information cues, benefits, health literacy, and health empowerment were assessed.

**Results:**

Perceived argument quality and perceived source credibility have significant and positive effects on perceived information benefits, but only perceived argument quality has a significant effect on perceived decision-making benefits. Two types of perceived benefits, in turn, affect health empowerment. The effects of perceived argument quality on perceived informational benefits and perceived decision-making benefits on health empowerment are significantly stronger for the high health literacy group than the low health literacy group (t_269_=7.156, *P*<.001; t_269_=23.240, *P*<.001). While, the effects of perceived source credibility on perceived informational benefits and perceived informational benefits on health empowerment are significantly weaker for the high health literacy group than the low health literacy group (t_269_=–10.497, *P*<.001; t_269_=–6.344, *P*<.001). The effect of perceived argument quality on perceived informational benefits shows no significant difference between high and low health literacy groups.

**Conclusions:**

In the context of online health information, perceived information benefits and perceived decision-making benefits are the antecedents of health empowerment, which in turn will be affected by perceived argument quality and perceived source credibility. Health literacy plays a moderating role in the relationship of some variables. To maximize health empowerment, online health information providers should strengthen information quality and provide differentiated information services based on users' health literacy.

## Introduction

### Background

Health empowerment is a cornerstone of a patient-centered approach to healthcare. Empowerment allows patients to take the initiative in making decisions about their own health care and quality of life, rather than passively complying with decisions made by others [1,2]. Previous literature hailed health empowerment as a new paradigm for health management and nursing practice [3,4]. As such, how to promote individuals’ health empowerment has become a common concern of scholars and health care professionals.

The rise of e-health services has brought new opportunities for promoting health empowerment. Various forms of electronic health services (eg, health information portals, online health communities, consultation platforms, etc) provide the public with abundant and easily accessible health information. Patients can obtain information about the symptoms of the disease, conventional treatment methods, and the treatment experience of others. With that health information, patients can become informed before doctors' visits and participate in health decision-making during the consultation process to enhance their sense of control. And an increasing number of people now obtain health information online. The China Internet Network Information Center pointed out that more than 276 million users in China utilize internet medical services, accounting for 29.4% of all internet users [5]. Online health information is changing the traditional way of the doctor visit, in which patients passively follow doctor's decisions.

Research on health empowerment in the context of eHealth services has become an important research stream. Some scholars explored the logic or dimensions of empowerment in the context of eHealth services [6,7]. Other scholars focused on the promotion of health empowerment by the benefits or functions of online health services. Electronic health records make users more informed and in a favorable position in the medical market [8]. Online health communities can provide users with various social support to promote empowerment [9-12]. Berkel et al [13] showed patients discussing drug use information on online message boards can promote patient empowerment, and the most common empowerment process is providing information and sharing personal experiences. Nelson et al [14] extracted the six system elements of wearable devices and pointed out that they can promote the user's health empowerment and commitment to health goals. In addition, some scholars have pointed out that web-based interventions can promote patient empowerment [15-17].

Previous research provided us with valuable knowledge for understanding health empowerment. Undoubtedly, obtaining health information from online resources to reduce information asymmetry is an indispensable part of patient empowerment [18,19]. However, perhaps we should pay more attention to the mechanism of information processing on health empowerment currently. Accessing online health information is easy nowadays due to high internet penetration, available devices, available information, but once information has been accessed, processing information is a crucial next step. In the context of processing online health information, individuals with different health literacy may face different situations. Health literacy measures the ability to acquire, process, and understand basic health information and the ability to use health information to make healthy decisions [20]. The usefulness of online health information largely depends on the recipients' health literacy [21]. Even in the face of the same online health information, the receivers with different health literacy will have different perceptions and health empowerment. In order to promote health empowerment more efficiently, it is necessary to focus further on information processing, explore how information recipients benefit from online health information, and ultimately promote health empowerment. Therefore, this study focuses on two issues: (1) How do users’ processing of health information contribute to health empowerment? (2) How does health literacy affect an individual's health empowerment process?

Overall, we assume that online health information can promote health empowerment during the consultation process, which is the result of the interaction information factors and the health literacy of the recipient. As a popular health resource, online health information can support patients with the ability to participate in the consultation process. Therefore, it is necessary to explore the process by which patients analyze online information and identify the mechanisms by which they contribute to health empowerment. To address this question, based on the elaboration likelihood model (ELM), we conceptualized perceived argument quality, perceived source credibility, and health literacy into online health information processing scenarios and explored their impact on health benefits and health empowerment.

### Literature Review

#### Health Empowerment

Empowerment theory has been explored by a rich body of research in social work, mainly as it relates to self-esteem, self-worth, self-confidence, and wellness [22,23]. Health empowerment is a further development of empowerment theory in the medical field. As part of a patient-centered philosophy, health empowerment emphasizes that individuals are responsible for their own health [24]. Health empowerment focuses on keeping individuals informed, encouraging active patient participation in decision-making [25,26], and working toward individual self-efficacy with regard to health matters [27,28].

Although health empowerment has been one of the core concepts in health promotion research, there is still no unified definition. Past researches have mainly defined health empowerment from three perspectives: process, emergent state, and active behavior [29]. From a process perspective, health empowerment is defined as the process leading to personal transformation, through which the individual’s ability to cope with health problems is developed [30,31]. In general, the implementation of the empowerment process requires the support of external resources. From an emergent state perspective, health empowerment represents the individual's health skills and psychological cognition, such as the health knowledge, skills, attitudes, and self-awareness with which people can make better health decisions [32]. This definition highlights motivation and ability and assesses an individual's state of being empowered. From an active behavior perspective, health empowerment is interpreted as the actual behavior change after possession, ability, and motivation.

Due to different definitions and research contexts, previous studies have used multidimensional or single-dimensional assessments of health empowerment. Ouschan et al [33] proposed that empowerment in the context of medical consultation includes three dimensions: patient control, patient participation, and doctor support. Prigge et al [34] understand empowerment as the behaviors that meet the inherent needs of autonomy and competence, including three dimensions: information search, knowledge development, and decision-making participation. From the perspective of the internal motivation process, Londono and Schulz [35] evaluate health empowerment in four dimensions: meaning, competence, self-determination, and impact. There are also some studies that assess health empowerment from a single dimension [36,37].

This study considers health empowerment from the perspective of the state of being empowered. Accessible online health information eases the information asymmetry between doctors and patients to a certain extent. The patient is no longer in a completely passive position but can actively participate in health activities. This undoubtedly allows patients to advocate for themselves and increase their sense of control. We define health empowerment as one's belief that they have a significant influence over health outcomes, including the ability to address personal health issues and feel in control over factors that can impact health outcomes.

#### Elaboration Likelihood Model

The elaboration likelihood model (ELM) explains how two types of information persuasion paths affect individuals’ attitude changes, perceptions, and behaviors [38]. The ELM has been used for many information systems literature as the theoretical basis for researching information adoption [39,40], online physician selection [41], and information technology adoption contexts [42,43]. The model postulates that external information can lead to attitude changes by two means: the central route and the peripheral route. The two routes distinguish one another in terms of the level of cognitive effort involved in processing information [38]. For the central route, persuasion results from careful consideration of the arguments regarding the core issues presented by the information. The recipients exert a high degree of cognitive effort. For the peripheral route, persuasion does not come from the information itself but from nonissue-related concerns, and the recipients devote less cognitive effort to the process [38,44]. The influence of each of the two routes can cause attitude changes and consequent behavior changes, but the changes caused by the central route are usually more stable and long-lasting than those caused by the peripheral route [45,46].

In addition, the ELM generally approaches elaboration likelihood from two influencing dimensions: ability and motivation [45]. If information recipients view a given message as being important or have a greater belief that they are capable of processing the information, they are more likely to invest the needed cognitive effort. In contrast, if recipients view the same message as having little personal relevance, or if they believe that as nonexperts, they have little choice but to depend on peripheral cues, they may be unwilling to spend much time and effort to scrutinize the information content [40]. Hence, ability and motivation are generally considered to moderate the relationship between two types of routes and perception changes [47]. Typically, ELM researchers have operationalized central route processing in terms of perceived argument quality and peripheral route processing in terms of perceived source credibility. Perceived argument quality measures whether the information content provides sufficient reasoning or support to prove the validity of key claims [48], while perceived source credibility measures the reliability and perceived acceptance of the information provider [49].

### Research Model and Hypotheses

#### The Influence of Perceived Informational Benefits and Perceived Decision-making Benefits on Health Empowerment

By providing online health information and educational opportunities, information and communication technology (ICT) can empower users to deal with health issues and engage in their own health outcomes [50,51]. Assessing consumers’ perceived benefits from the use of ICT can enable health professionals and researchers to develop better strategies for using ICT as an empowerment tool to support users in accessing information and managing health. Perceived informational benefits reflect the users’ ability to better understand their own health status and treatment options with the support of online information. Perceived decision-making benefits measure the extent to which users can participate effectively in decision-making for their own well-being with the help of knowledge or experiences obtained from the internet [26]. Health empowerment is based on the premise that the individual can obtain relevant medical knowledge and skills [52]. The availability of online health information allows users to acquire the knowledge and skills they need to enhance their self-efficacy. This knowledge allows them to be more confident about participating in treatment decisions by addressing questions to their physicians, sharing feelings, and otherwise being actively involved in their own health care [53].

Online support groups enable patients to learn more about themselves, enhance their social well-being, and thus promote healthy empowerment [11]. Johnston et al [19] explored the impact of participation in online health communities on health empowerment from the perspective of information utility. Their findings showed that online health communities could provide participants with direct benefits such as practical information and social support to further promote their health empowerment.

Since involvement in health consultation and decision-making processes is an important element of health empowerment [54], individual participation in the medical decision-making process will help patients understand medical practices, maximize individual satisfaction, and achieve a better quality of care [55,56]. Health empowerment can be improved by developing individuals’ ability to participate actively in the medical decision-making process [57]. With the increasing availability of online health information, individuals can better interact with their physicians, evaluate services more accurately, and make informed decisions. Therefore, we proposed the following hypotheses:

- **H1:** Perceived informational benefits have a positive impact on health empowerment.
- **H2:** Perceived decision-making benefits have a positive impact on health empowerment.

#### The Influence of Perceived Argument Quality on Perceived Benefits

Perceived argument quality is reflected in an individual's subjective evaluation of the reasoning that forms the core of presented information. The presentation of information can be strong and convincing or weak and specious. Strong arguments mean that the presented information is reasonable and convincing to the recipient, while weak arguments are doubtful or contradictory [38,58]. In the ELM, perceived argument quality that follows the central path of cognitive processing is an important factor affecting attitudes and decision-making [45,59]. In the information adoption model proposed by Sussman and Siegal [40], perceived argument quality was used as a predictor of the perceived usefulness of the information. Their empirical results showed that as the quality of information arguments increased, the perceived usefulness and adoption intention increased as well. In health information research, the literature has pointed out that perceived argument quality exerts an influence on recipients’ attitude changes and is an important index used to evaluate the quality of information [44,60]. The quality of information is directly related to whether information seekers can obtain the information they need and make high-quality decisions [61]. Therefore, perceived argument quality will affect the individual’s perceived informational and decision-related benefits. When confronted with the uneven quality of online health information, the recipients can judge the quality and usefulness of the information according to the quality of the arguments to obtain complete, accurate, and validated online health information. In line with the idea that quality information can help people better understand their own health status and perform well in making health decisions, the following hypotheses were proposed:

- **H3a:** The perceived argument quality has a positive impact on the perceived informational benefits.
- **H3b:** The perceived argument quality has a positive impact on the perceived decision-making benefits.

#### The Influence of Perceived Source Credibility on Perceived Benefits

Perceived source credibility is the evaluation of information from the reliability of information sources. It can be perceived to be credible, acceptable, or untrustworthy by information recipients [62]. A highly credible information source is more persuasive than a less creditable one [49,63]. In the ELM, perceived source credibility as a peripheral route factor affects the attitude change of the information recipient [45,59]. In the context of information adoption, perceived source credibility has been identified as peripheral clues of the given messages that affect information’s usefulness [40]. In the context of consumer-to-consumer communication, the credibility of information derived from communication can help consumers evaluate the quality of products, allowing the consumers to make reasonable purchase decisions [64].

In the health information literature, perceived source credibility is an important topic that relates to individual health outcomes and decision-making behavior. Young people's trust in health information is affected by perceived source credibility. The higher credibility of the information source, the more likely the users are to participate in the information activity [65,66]. Ghaddar et al [67] demonstrated that exposure to credible sources of health information could improve individual health literacy. High-quality health information is the basis for individuals to improve their health knowledge and participate in treatment decisions. In addition, source credibility is an important factor used by individuals to evaluate the quality of online health information [68,69]. When faced with uncertain quality online health information, highly credible sources can reduce perceived risk and increase trust in health information [44]. Individuals who obtain more credible health information can better understand their own health conditions and participate more effectively in health decision-making processes, whereas individuals who possess unreliable health information may be led to negative outcomes [70]. Hence, we hypothesized:

- **H4a:** The credibility of sources has a positive effect on the perceived informational benefits.
- **H4b:** The credibility of sources has a positive effect on the perceived decision-making benefits.

#### Moderating Effect of Health Literacy

According to the ELM, the ability to process information can affect the level of elaboration likelihood [45]. In our work, health literacy was identified to measure ones’ ability to process health information gained online. Health literacy measures the ability to acquire, process, and understand basic health information, as well as the ability to use health information to make healthy decisions [20]. Previous research demonstrated that health literacy correlates with individuals’ health information acquisition and use behaviors. Individuals with adequate health literacy are more inclined to access health information through multiple channels, such as the internet, rather than relying solely on medical personnel [71,72]. Ghaddar et al [67] also pointed out that health literacy positively affects the self-efficacy and motivation of individuals to gather the information needed from online sources. In addition, health literacy determines the effective response of information recipients to health information to a certain extent. Individuals with limited health literacy can obtain more information as needed if the information providers reduce the cognitive requirements for understanding online information [21].

In the process of health information analysis, individuals with adequate health literacy have a greater ability to analyze the arguments presented as part of health information. For individuals whose attitudes or perceptions change based on central route processing, the information influence occurs under conditions of high-end elaboration (ie, content-oriented reasoning). In contrast, for individuals with limited health literacy, information processing is more about evaluating factors other than content, so peripheral cues play a more critical role in processing. These expectations led us to state the following hypotheses:

- **H5a:** For users with high health literacy, perceived argument quality has a stronger impact on perceived informational benefits than that of users with low health literacy.
- **H5b:** For users with high health literacy, perceived argument quality has a stronger impact on perceived decision-making benefits than that of users with low health literacy.
- **H6a:** For users with low health literacy, perceived source credibility has a stronger impact on perceived informational benefits than that of users with high health literacy.
- **H6b:** For users with low health literacy, perceived source credibility has a stronger impact on perceived decision-making benefits than that of users with high health literacy.

We assume that the perceived informational benefits and decision-making benefits all contribute to empowerment. However, the two kinds of benefits have different requirements for patients’ health literacy. Informational benefits are the prerequisite for decision-making benefits. Sufficient information can improve the quality of decision-making and reduce risks [73]. Meanwhile, decision-making requires patients to devote more cognitive costs. Because decision-making means that patients have to make some trade-offs, such as choosing a suitable therapy among multiple treatment options[74], these require patients to organize and process health information at a deeper level, which means that adequate health literacy is needed. Individuals with high health literacy tend to participate in health decisions [74,75]. Therefore, patients have varying degrees of demands or expectations for informational benefits and decision-making benefits in promoting empowerment. For groups with high health literacy, the appeal of rights and interests in the consultation is not limited to getting more information but also encourages participation in the decision-making process. For groups with low health literacy, their information processing capacity is insufficient to form effective health decisions; their appeals focus on obtaining informational benefits. Therefore, we get the following hypotheses:

- **H7a:** For users with high health literacy, perceived informational benefits have a weaker impact on health empowerment than that of users with low health literacy.
- **H7b:** For users with high health literacy, perceived decision-making benefits have a stronger impact on health empowerment than that of users with low health literacy.

**Figure 1:**
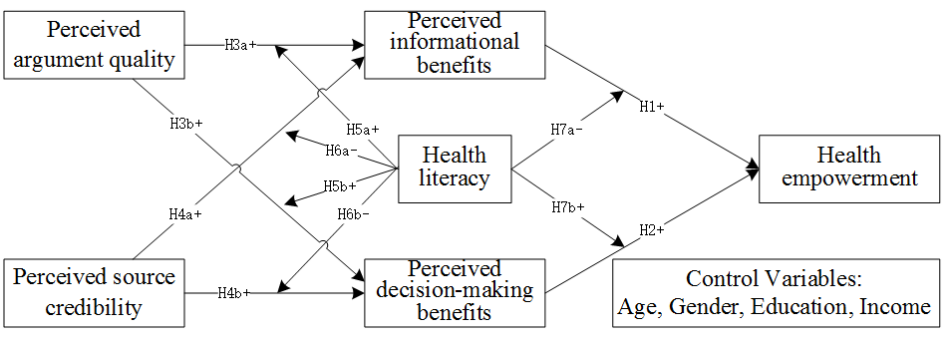
Research model.

A summary of the conceptual research model is depicted in Figure 1.

## Methods

### Measurement Development

To test our hypotheses, we administered a self-reported questionnaire to collect data. The questionnaire consisted of two parts: one was designed to investigate the demographic characteristics of the participants, and the other focused on the measurement of the constructs. The research model contained a total of 6 constructs. The measurement scales were developed by drawing on prior literature, and some items were fine-tuned according to the background of this study. We adapted the work of Hur et al [76] to measure perceived argument quality (eg, “the health information provided online is informative” and “the health information provided online is persuasive”) and the work of Sussman and Siegal [40] to measure perceived source credibility (eg, “the provider of online health information is knowledgeable” and “the provider is an expert on the message topic”). The measurement of perceived informational benefits (eg, “by searching for online health information, I feel better informed as a patient” and “by searching for online health information, I understand my illness better” ) was adapted from the paper by van Uden-Kraan et al [11], and the measurement of perceived decision-making benefits (eg, “online health information is helpful to decide what questions to ask during doctor appointments” and “online health information is helpful to decide on treatment choices and make decisions” ) was adapted from Seçkin [26]. The determination of health literacy (eg, “I know how to use the internet to answer my questions about health” and “I have the skills I need to evaluate the health resources and information I find on the internet” ) was adapted from the eHealth Literacy Scale [77]. The items used to measure health empowerment (eg, “I feel more in control of my health” and “I know what to do to take care of my health problem”) were adapted from Bann et al [36]. There are two reasons for the choice. First, the content captured by the empowerment in this study is similar to Bann et al [36], that is, enablement and the sense of control. Although the two research contexts are different, both explore the improvement of patients' ability and a sense of control with the support of external convenience. Second, the scale has been used in many studies [78-80], and it has been proven to have good reliability and validity. For all constructs, measures were designed using 5-point Likert scales from 1 (“strongly disagree”) to 5 (“strongly agree”).

Since the respondents are Chinese, we need to translate all the items from English into Chinese. All measures were back-translated by another translator who did not know the background of the study to ensure the accuracy of the translation. The two English versions were compared, and potential semantic discrepancies were examined to ensure that the Chinese scales reflected the meaning of all measures accurately. Then 10 postgraduates with experience seeking online health information were invited to participate in a pretest of the scales. Based on their feedback, any ambiguous expressions were amended. The measured constructs and their sources are shown in Multimedia Appendix 1.

### Survey Administration

We collected data through a questionnaire service website [81]. The survey participants were required to have had experience seeking online health information before they consulted a doctor during the most recent 6-month period to ensure that participants had an accurate understanding of each measurement item. In addition, subjects were asked to evaluate each measurement item based on their last used experience. A total of 371 questionnaires were collected within 2 weeks. We deleted 28 uncompleted or invalid questionnaires, leaving a total of 343 valid responses. The response rate was 92.7%.

Among the valid questionnaires, 47.2% (162/343) were from males, and 52.8% (181/343) were from females. Further, 85.1% (292/343) of respondents’ ages ranged from 18-35 years, implying that the majority of online health information users tend to be younger. In terms of education, 88.9% (305/343) of the respondents had a college degree or above. The majority (234/343, 68.2%) of the respondents had a monthly disposable income in the range of 3000-8999 Chinese Yuan (approximately US $469-1406). As to their occupations, business employees accounted for the largest proportion of participants, reaching 47.5% (163/343). The most popular way to access information was through a health information portal, accounting for 61.8% (212/343) of the respondents, followed by a health consulting platform, accounting for 22.7% (78/343). On average, 63.3% (217/343) of the respondents used online health information sources between 1 and 3 times weekly, and 22.4% (77/343) of the subjects used these sources 4 to 5 times weekly. The specific demographic information of the target samples is shown in Table 1.

**Table 1:** Demographic information of respondents (N=343).

Characteristics			Participants, n (%)
**Gender**			
	Male	162(47.2)	
	Female	181(52.8)	
**Age, years**			
	18-25	89(25.9)	
	26-35	203(59.2)	
	36-45	45(13.2)	
	46 and above	6(1.7)	
**Education**			
	High school or below	38(11.1)	
	Associate degree	101(29.4)	
	College degree	176(51.3)	
	Master degree or above	28(8.2)	
**Income, Chinese Yuan^a^/month**			
	Under 3000	83(24.2)	
	3000—5999	148(43.1)	
	6000—8999	86(25.1)	
	9000—11,999	16(4.7)	
	12,000 and above	10(2.9)	
**Occupation**			
	Student	41(12)	
	Business employees	163(47.5)	
	Government and public institutions	39(11.4)	
	Self-employed persons	45(13.1)	
	Other	55(16)	
**Weekly usage frequency (times)**			
	1-3	217(63.3)	
	4-5	77(22.4)	
	6-7	25(7.3)	
	7 and above	24(7)	
**Information channel**			
	Health information portal	212(61.8)	
	Online patient community	36(10.5)	
	Health consultation platform	78(22.7)	
	Blog or video	8(2.3)	
	Other	9(2.6)	

^a^A currency exchange rate of ¥1 = US $0.16 is applicable.

## Results

### Overview

We used variance-based partial least squares structural equation modeling (PLS-SEM) for data analysis. We chose the PLS-SEM method for the following reasons. First, the PLS-SEM method does not require multivariate normal distribution data [82]. We performed Kolmogorov-Smirnov test (K-S test) to examine the distribution of sample data. And we found that the significance level of all items is less than 0.05. Therefore, the null hypothesis is rejected, meaning the data is nonnormally distributed. Second, PLS-SEM is suitable for exploratory research because it aims at theoretical development rather than the confirmation of the established theory [82,83]. Finally, the PLS-SEM method has fewer restrictions on the sample size [84]. Compared with other methods, it can obtain greater statistical power with nonlarge sample size. We first examined the measurement model and then the structural model.

### Reliability and Validity Analysis

In this study, we used the confirmatory factor analysis process to test the measurement model. As shown in Table 2, Cronbach’s α of all constructs is between 0.717 and 0.895. And the composite reliability of each construct is between 0.823 and 0.916. These are above the recommended value of 0.7, which means that the measurement model has good reliability [85]. To assess convergent validity, we measured the standard loading of each item as well as the average variance extracted (AVE) for each construct. The results showed that the items’ loadings range from 0.678 to 0.833. Among them, two items’ loadings (PAQ4 and PDB1) are less than 0.7 but still much larger than the cutoff value of 0.6 [86]. Also, the AVE of each construct surpasses 0.5. These results imply that the measurement model has good convergence validity [85].

Furthermore, as shown in Table 3, the square root of the AVE of each construct is larger than its correlation coefficients with other constructs, which means the discriminant validity of the measurement model is confirmed [85].

**Table 2:** Results of confirmatory factor analysis.

Construct and item		Loading	Cronbach’s α	Composite reliability	AVE^a^
**Perceived argument quality (PAQ)**			0.764	0.850	0.587
	PAQ1	0.750			
	PAQ2	0.827			
	PAQ3	0.784			
	PAQ4	0.695			
**Perceived source credibility (PSC)**			0.802	0.870	0.627
	PSC1	0.785			
	PSC2	0.781			
	PSC3	0.793			
	PSC4	0.808			
**Perceived informational benefits (PIB)**			0.756	0.845	0.578
	PIB1	0.787			
	PIB2	0.747			
	PIB3	0.708			
	PIB4	0.797			
**Perception decision-making benefits (PDB)**			0.717	0.823	0.539
	PDB1	0.678			
	PDB2	0.762			
	PDB3	0.724			
	PDB4	0.769			
**Health empowerment (EM)**			0.786	0.854	0.539
	EM1	0.736			
	EM2	0.775			
	EM3	0.702			
	EM4	0.736			
	EM5	0.720			
**Health literacy (HL)**			0.895	0.916	0.578
	HL1	0.773			
	HL2	0.833			
	HL3	0.787			
	HL4	0.712			
	HL5	0.715			
	HL6	0.758			
	HL7	0.750			
	HL8	0.747			

^a^AVE: average variance extracted.

**Table 3:** Means, SD, and correlation matrix.

Variable	Mean	SD	PAQ^a^	PSC^b^	PIB^c^	PDB^d^	EM^e^	HL^f^
PAQ	3.910	0.680	**0.766**	—^g^	—	—	—	—
PSC	3.625	0.725	0.624	**0.701**	—	—	—	—
PIB	3.918	0.722	0.634	0.553	**0.760**	—	—	—
PDB	3.812	0.656	0.514	0.409	0.632	**0.734**	—	—
EM	3.676	0.651	0.424	0.410	0.454	0.402	**0.734**	—
HL	3.433	0.799	0.569	0.550	0.593	0.553	0.603	**0.760**

^a^PAQ: perceived argument quality.

^b^PSC: perceived source credibility.

^c^PIB: perceived informational benefits.

^d^PDB: perceived decision-making benefits.

^e^EM: health empowerment.

^f^HL: health literacy.

^g^—: The correlation matrix is symmetrical; therefore, only the lower-left corner is displayed.

As our data were collected from single respondents, common method variance (CMV) may threaten the validity of the results. To test such bias, first, we used Harman’s single-factor test to assess the 6 constructs in the search model. The results showed that the variance explained by the first factor is 35.4%, which does not exceed 50% [87]. Second, we verified the issue by using the potential factor method from Liang et al [88]. We introduced a common method factor into the PLS-SEM model, which contains all the constructs’ indicators. Then we calculated to what extent the common method factor and the main constructs explain the variance of each indicator, respectively. As shown in Multimedia Appendix 2, the average explain variance based on major constructs and the common method factor is 0.579 and 0.006, respectively, with a larger ratio between them. In summary, CMV should not be a serious concern for this study.

### Test of Main Effects

**Figure 2:**
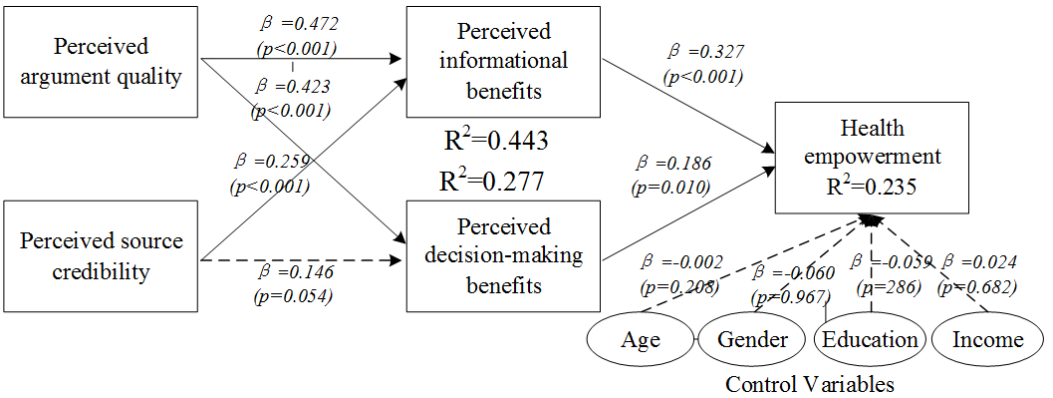
PLS Analysis of main effects. PLS: partial least squares.

In this paper, we used SmartPLS 3.0 (SmartPLS GmbH) to test the research model. The path coefficients and significance levels of main effects are shown in Figure 2. Perceived informational benefits (β=.327, *P*<.001) and perceived decision-making benefits (β=.186, *P*=.01) exerted positive effects on users’ health empowerment, indicating that H1 and H2 were supported. Perceived argument quality had a positive effect on perceived informational benefits (β=.472, *P*<.001) and on perceived decision-making benefits (β=.423, *P*<.001), indicating that H3a and H3b were supported. Perceived source credibility had a positive effect on perceived informational benefits (β=.259, *P*<.001), but the effect on perceived decision-making benefits was not significant. Thus, hypothesis H4a was supported, but H4b was not supported. All control variables had no significant effect on health empowerment. The variances explained by perceived informational benefits, perceived decision-making benefits, and healthy empowerment were 44.3%, 27.7%, and 23.5%, respectively.

### Test of Moderating Effects

A multigroup comparison method developed by Keil et al [89] was used to test the moderating effect of health literacy. This method tests the moderating effect by evaluating the difference in path coefficients between subgroups and has been used in many studies [90,91]. We first divided the samples into high health literacy and low health literacy groups by the median (3.75), with sample sizes of 174 (≥3.75) and 169 (<3.75). Then we used the data of each subgroup to test the research model and compared the path coefficients.

As shown in Table 4, the path coefficients from perceived argument quality to perceived informational benefits and perceived decision-making benefits to empowerment are significantly stronger for the high health literacy group than the low health literacy group (t_341_=7.156, *P*<.001; t_341_=23.240, *P*<.001). The path coefficients from perceived source credibility to perceived informational benefits and from perceived informational benefits to empowerment are significantly weaker for the high health literacy group than the low health literacy group (t_341_=–10.497, *P*<.001; t_341_=–6.344, *P*<.001). Therefore, H5a, H6a, H7a, and H7b were supported. The path coefficients from perceived argument quality to decision-making benefits show no significant difference between the two subgroups. Therefore, H5b was not supported. Although path coefficients perceived source credibility to perceived decision-making benefits show a significant difference, they are not significant in either group.

**Table 4:** The results of moderating effects.

Paths	High health literacy (n=174)		Low health literacy (n=169)		t_341_ values comparing the two groups
	Coefficient	SE	Coefficient	SE	
PAQ^a^→PIB	0.464	0.099	0.395	0.078	7.156
PAQ→PDB	0.358	0.107	0.341	0.099	1.526
PSC^b^→ PIB	0.181	0.076	0.27	0.081	–10.497
PSC→PDB	0.067	0.122	0.151	0.102	–6.908
PIB^c^→EM^d^	0.190	0.094	0.266	0.126	–6.344
PDB^e^→EM	0.292	0.089	–0.003	0.141	23.240

^a^PAQ: perceived argument quality.

^b^PSC: perceived source credibility.

^c^PIB: perceived informational benefits.

^d^EM: health empowerment.

^e^PDB: perceived decision-making benefits.

### Test of Mediating Effects

The bootstrap method was used for the analysis of mediating effects [92]. This method can directly test the indirect effects of independent variables on the dependent variables and does not require the mediating effects to follow normal distribution [93]. Using SmartPLS 3.0, we performed bootstrap with 5000 resamples to obtain a 95% CI for indirect effects and direct effect. According to the results in Table 5, the direct effect of perceived argument quality on health empowerment was not significant. Meanwhile, the indirect effects of perceived argument quality on health empowerment (ie, PAQ→PIB→EM and PAQ→PDB→EM) were significant. It means that perceived informational benefits and perceived decision-making benefits play a fully mediating role between perceived argument quality and health empowerment. The direct effect of perceived source credibility on health empowerment was significant, and the indirect effect of the two variables (ie, PSC→PIB→EM) was also significant. This means that the effect of perceived source credibility on health empowerment was partially mediated by perceived informational benefits. With perceived decision-making benefits as the mediate variable, the indirect effect of PSC on health empowerment was not significant, indicating that perceived decision-making benefits have a nonmediating role in the effect of perceived source credibility on health empowerment.

**Table 5:** The results of the mediation effect test.

Indirect path	95%CI	Direct path	95%CI	Result
PAQ^a^→PIB^b^→EM^c^	0.007 to 0.173	PAQ→EM	–0.011 to 0.256	Full
PAQ→PDB^d^→EM	0.005 to 0.129	PAQ→EM	–0.011 to 0.256	Full
PSC^e^→PIB→EM	0.004 to 0.097	PSC→EM	0.058 to 0.285	Partial
PSC→PDB→EM	–0.002 to 0.061	PSC→EM	0.058 to 0.285	None

^a^PAQ: perceived argument quality.

^b^PIB: perceived informational benefits.

^c^EM: health empowerment.

^d^PDB: perceived decision-making benefits.

^e^PSC: perceived source credibility.

## Discussion

### Principal Findings

Based on the ELM model, this paper examined the influencing factors of health empowerment in the context of processing online health information. Our empirical research provided the following results:

First, perceived informational and decision-making benefits are important predictors of users’ health empowerment. Perceived informational benefits accrue when individuals become more informed by browsing online health information. This input allows them to have a more objective understanding of their illnesses and health situations, thereby reducing negative emotions, such as anxiety and panic. Perceived decision-making benefits refer to growth in terms of knowledge and skills gained through seeking online health information. This improvement in decision-making capacity allows individuals to participate more effectively in the consultation process and make reasonable suggestions for treatment. The gain of these two kinds of benefits makes users feel empowered.

Second, the results confirm that perceived argument quality, as involved with the central route, has a positive effect on perceived informational and decision-making benefits, while perceived source credibility, which relies on the peripheral route, only has a significant impact on perceived informational benefits. When getting health information from online channels, the strength of the arguments and credibility of the sources reflect the quality of information. They are the guarantee that users can benefit from the information they receive. Both high-quality arguments and credible sources can enhance an individual's acceptance and approval of the information, thus promoting the perceived informational benefits. Individuals need knowledge and skills to make informed health decisions. The online health information presented with high-quality arguments can provide recipients with health knowledge and treatment experience so they can make informed decisions in medical consultations.

Our results show that the credibility of sources has no significant influence on perceived decision-making benefits. One possible explanation is that the basis for supporting individuals’ participation in decision-making may come more from the information itself, which develops individuals’ knowledge or skills. However, the credibility of information resources as a peripheral cue does not improve knowledge or skill levels and thus cannot support the individual’s participation in the decision-making process.

Third, we also found that the effects of the central route and the peripheral route are different in low and high health literacy groups. For individuals with high health literacy, the effect of central route processing (perceived argument quality) on perceived informational benefits is stronger than the influence of processing using peripheral cues (perceived source credibility). Individuals with high health literacy are more likely to exert cognitive effort when assessing the arguments provided by online information. For these individuals, information source credibility is used as a secondary consideration and has a weaker effect on perceived informational benefits. The opposite is true for individuals with low health literacy. For low health literacy groups, their judgments of online health information rely more on the source credibility.

Finally, the study demonstrates that the effects of the two perceived benefits on health empowerment are different between groups with high and low health literacy. The effect of perceived informational benefits on health empowerment is greater in the low health literacy group than in the high health literacy group. The effect of perceived decision-making benefits on health empowerment is significant in the high health literacy group but not in the low-health literacy group. The results show that there is a higher demand for health empowerment for individuals with high health literacy. Merely information benefits are not enough to promote health empowerment but to further obtain perceived decision-making benefits. For low health literacy groups, health empowerment does not derive from participating in decision-making but from getting enough information to reduce information asymmetry.

### Theoretical Implications

There are two theoretical contributions of this study. First, the study provided a profound understanding of the mechanism of health information processing on health empowerment. Previous studies highlighted the convenience and positive health outcomes that can be derived from information technology and online health information [9,10,14]. However, fewer studies have focused on how the process of information processing influence users’ perceived benefits and health empowerment. To advance this line of research, this study explored how the two information processing routes affect the individuals’ perceived benefits and health empowerment. In this way, our work enriches the existing research on health empowerment promotion.

Second, this study explained the relationship between health literacy and health empowerment from a new perspective that is different from previous literature, which always explores the direct relationship between health literacy and health empowerment [94-96]. This study found that individuals with different health literacy have differences in the processing of online health information. Individuals with higher health literacy tend to focus on the central route, while those with lower health literacy focus on peripheral cues. The findings of this study provide a new perspective for studying the relationship between health literacy and health empowerment in other contexts.

### Practical Implications

Based on our theoretical analysis and empirical results, the following practical implications should be noted. First, encouraging patients to search for high-quality online health information is an effective way to promote their health empowerment. The information provider can strengthen information quality management in terms of perceived argument quality and perceived source credibility. Accordingly, to prevent the dissemination of misleading content, online health information providers should establish reasonable evaluation and testing mechanisms. They should strictly scrutinize every piece of health information provided to consumers and ensure that information content is complete, rigorous, sound, and scientific.

Second, information providers should also consider the health literacy of recipients while providing health information. To improve the effectiveness of promoting health empowerment, online health information providers should establish a health literacy assessment mechanism to provide targeted information services to individuals with different health literacy. For individuals with a high level of health literacy, it is an effective strategy to cultivate users' health decision-making ability to promote health empowerment, and providers should highlight the scientific nature of the information. For those with a lower level of health literacy, making them more informed is an effective way to promote empowerment, and providers should highlight the professionalism and reliability of the sources of information.

### Limitations of the Study

Although this paper draws some conclusions that cannot be ignored, there are still some shortcomings that should be addressed in the future. First, this study did not consider the impact of the type of online health information service model used by consumers to gather information, such as an online health consultation website or a medical information portal. The unique characteristics of different online health information services may impact health empowerment. Second, our study was based on a static model and cross-sectional data. The processes that affect the promotion of individual health empowerment are likely to be dynamic, so longitudinal research is necessary. Third, we did not involve the measurement of the respondent’s disease and pathology, which may affect a person’s use of online health information. Finally, medical consultation is a process of interaction between patients and doctors. This research only focuses on patient factors. Future research should consider the impact of doctor-related factors (such as empathy and patient-centered communication) on health empowerment.

### Conclusion

In this paper, we explored the effect of the central route and peripheral route of online health information on users’ health empowerment. We also considered the moderating role of health literacy in both routes. To test the hypothesis, PLS-SEM was used to analyze the data, and the empirical results supported most of the hypothesis. The findings further confirmed the important role of electronic information technology in promoting health empowerment. In the context of online health information, we must pay more attention to information quality and the interaction effect between individuals’ health literacy and information processing cues. Research results provide practical guidance for health information providers to better serve and maximize individuals’ benefits and empowerment. This study also pointed out the differences in promoting health empowerment besides health literacy. And more research in the future is needed to focus on individualized differences in the promotion of health empowerment.

## Appendix

Multimedia Appendix 1 Measurement Scales.

Multimedia Appendix 2 Common Method Bias Analysis.

## Abbreviations

AVE: average variance extracted
CMV: common method variance
ELM: elaboration likelihood model
ICT: information and communication technology
PLS-SEM: partial least squares structural equation modeling

## References

[1] Castro EM, Van Regenmortel T, Vanhaecht K, Sermeus W, Van Hecke Ann (2016). Patient empowerment, patient participation and patient-centeredness in hospital care: A concept analysis based on a literature review. Patient Educ Couns.

[2] Feste C, Anderson RM (1995). Empowerment: from philosophy to practice. Patient Educ Couns.

[3] Anderson RM, Funnell MM (1999). Theory is the cart, vision is the horse: reflections on research in diabetes patient education. Diabetes Educ.

[4] Anderson RM, Funnell MM (2005). Patient empowerment: reflections on the challenge of fostering the adoption of a new paradigm. Patient Educ Couns.

[5] (2020). The 46th China Statistical Report on Internet Development. CNNIC.

[6] Almunawar M, Anshari M, Younis MZ (2015). Incorporating customer empowerment in mobile health. Health Policy and Technology.

[7] Lemire M (2010). What can be expected of information and communication technologies in terms of patient empowerment in health?. J Health Organ Manag.

[8] Vezyridis P, Timmons S (2015). On the adoption of personal health records: some problematic issues for patient empowerment. Ethics Inf Technol.

[9] Audrain-Pontevia A, Menvielle L (2018). Do online health communities enhance patient-physician relationship? An assessment of the impact of social support and patient empowerment. Health Serv Manage Res.

[10] Sharma S, Khadka A (2019). Role of empowerment and sense of community on online social health support group. ITP.

[11] van Uden-Kraan CF, Drossaert CHC, Taal E, Seydel ER, van de Laar MAFJ (2009). Participation in online patient support groups endorses patients' empowerment. Patient Educ Couns.

[12] Petrič G, Atanasova S, Kamin T (2017). Impact of Social Processes in Online Health Communities on Patient Empowerment in Relationship With the Physician: Emergence of Functional and Dysfunctional Empowerment. J Med Internet Res.

[13] van Berkel JJ, Lambooij MS, Hegger I (2015). Empowerment of patients in online discussions about medicine use. BMC Med Inform Decis Mak.

[14] Nelson EC, Verhagen T, Noordzij ML (2016). Health empowerment through activity trackers: An empirical smart wristband study. Computers in Human Behavior.

[15] van den Berg SW, Gielissen MFM, Ottevanger PB, Prins JB (2012). Rationale of the BREAst cancer e-healTH [BREATH] multicentre randomised controlled trial: an internet-based self-management intervention to foster adjustment after curative breast cancer by decreasing distress and increasing empowerment. BMC Cancer.

[16] Karni L, Dalal K, Memedi M, Kalra D, Klein GO (2020). Information and Communications Technology-Based Interventions Targeting Patient Empowerment: Framework Development. J Med Internet Res.

[17] McGloin H, O'Connell D, Glacken M, McSharry P, Healy D, Winters-O'Donnell L, Crerand K, Gavaghan A, Doherty L (2020). Patient Empowerment Using Electronic Telemonitoring With Telephone Support in the Transition to Insulin Therapy in Adults With Type 2 Diabetes: Observational, Pre-Post, Mixed Methods Study. J Med Internet Res.

[18] Sanders K, Sánchez Valle M, Viñaras M, Llorente C (2015). Do we trust and are we empowered by “Dr. Google”? Older Spaniards’ uses and views of digital healthcare communication. Public Relations Review.

[19] Johnston AC, Worrell JL, Di Gangi PM, Wasko M (2013). Online health communities: An assessment of the influence of participation on patient empowerment outcomes. Info Technology & People.

[20] Pecukonis E (2008). Health literacy: A prescription to end confusion. Social Work in Health Care.

[21] Meppelink CS, Smit EG, Diviani N, Van Weert JCM (2016). Health Literacy and Online Health Information Processing: Unraveling the Underlying Mechanisms. J Health Commun.

[22] Gibson CH (1991). A concept analysis of empowerment. J Adv Nurs.

[23] Nyatanga L, Dann KL (2002). Empowerment in nursing: the role of philosophical and psychological factors. Nursing Philosophy.

[24] Labonte R (1989). Community and professional empowerment. Can Nurse.

[25] Anderson RM, Funnell MM (2010). Patient empowerment: myths and misconceptions. Patient Educ Couns.

[26] Seçkin G (2010). Cyber patients surfing the medical web: Computer-mediated medical knowledge and perceived benefits. Computers in Human Behavior.

[27] Lee Y, Shin S, Wang R, Lin K, Lee Y, Wang Y (2016). Pathways of empowerment perceptions, health literacy, self-efficacy, and self-care behaviors to glycemic control in patients with type 2 diabetes mellitus. Patient Educ Couns.

[28] Moattari M, Ebrahimi M, Sharifi N, Rouzbeh J (2012). The effect of empowerment on the self-efficacy, quality of life and clinical and laboratory indicators of patients treated with hemodialysis: a randomized controlled trial. Health Qual Life Outcomes.

[29] Fumagalli LP, Radaelli G, Lettieri E, Bertele' P, Masella C (2015). Patient Empowerment and its neighbours: clarifying the boundaries and their mutual relationships. Health Policy.

[30] Funnell MM, Anderson RM (2004). Empowerment and Self-Management of Diabetes. Clinical Diabetes.

[31] Newton P, Sasha S, Koula A (2011). Marrying contradictions: healthcare professionals perceptions of empowerment in the care of people with Type 2 Diabetes. Patient Educ Couns.

[32] Funnell MM, Anderson RM, Arnold MS, Barr PA, Donnelly M, Johnson PD, Taylor-Moon D, White NH (1991). Empowerment: an idea whose time has come in diabetes education. Diabetes Educ.

[33] Ouschan R, Sweeney J, Johnson L (2006). Customer empowerment and relationship outcomes in healthcare consultations. European Journal of Marketing.

[34] Prigge J, Dietz B, Homburg C, Hoyer WD, Burton JL (2015). Patient empowerment: A cross-disease exploration of antecedents and consequences. International Journal of Research in Marketing.

[35] Londoño AMM, Schulz PJ (2015). Influences of health literacy, judgment skills, and empowerment on asthma self-management practices. Patient Educ Couns.

[36] Bann CM, Sirois FM, Walsh EG (2010). Provider support in complementary and alternative medicine: exploring the role of patient empowerment. J Altern Complement Med.

[37] Kim SC, Kim S, Boren D (2008). The quality of therapeutic alliance between patient and provider predicts general satisfaction. Mil Med.

[38] Petty RE, Cacioppo JT, Goldman R (1981). Personal involvement as a determinant of argument-based persuasion. Journal of Personality and Social Psychology.

[39] Watts S, Zhang W (2008). Capitalizing on Content: Information Adoption in Two Online communities. JAIS.

[40] Sussman SW, Siegal WS (2003). Informational Influence in Organizations: An Integrated Approach to Knowledge Adoption. Information Systems Research.

[41] Cao X, Liu Y, Zhu Z, Hu J, Chen X (2017). Online selection of a physician by patients: Empirical study from elaboration likelihood perspective. Computers in Human Behavior.

[42] Angst, Agarwal (2009). Adoption of Electronic Health Records in the Presence of Privacy Concerns: The Elaboration Likelihood Model and Individual Persuasion. MIS Quarterly.

[43] Bhattacherjee, Sanford (2006). Influence Processes for Information Technology Acceptance: An Elaboration Likelihood Model. MIS Quarterly.

[44] Yi MY, Yoon JJ, Davis JM, Lee T (2013). Untangling the antecedents of initial trust in Web-based health information: The roles of argument quality, source expertise, and user perceptions of information quality and risk. Decision Support Systems.

[45] Marquart F, Naderer B, Potthoff M (2016). Communication and Persuasion: Central and Peripheral Routes to Attitude Change. Schlüsselwerke der Medienwirkungsforschung.

[46] Wang HC, Doong HS, Shih HC, Pallister J, Foxall G (2008). An Investigation into the determinants of repurchase loyalty in the E-Marketplace.

[47] Koo C, Wati Y (2011). E-Healthcare Service: An Investigation of the Antecedents, Moderating Roles, and Consequences.

[48] Boller GW, Swasy JL, Munch JM (1990). Conceptualizing argument quality via argument structure. Advances in Consumer Research.

[49] Pornpitakpan C (2004). The Persuasiveness of Source Credibility: A Critical Review of Five Decades' Evidence. J Appl Social Pyschol.

[50] Harrison JP, Lee A (2006). The role of e-Health in the changng health care environment. Nurs Econ.

[51] Zaphiris P, Ang CS (2009). Social Computing and Virtual Communities.

[52] Watanabe N, Kaneko A, Yamar S, Taleo G, Tanihata T, Lum JK, Larson PS, Shearer NBC (2015). A prescription for sustaining community engagement in malaria elimination on Aneityum Island, Vanuatu: an application of Health Empowerment Theory. Malar J.

[53] Fleisher L, Bass S, Ruzek SB, McKeown-Conn N (2002). Relationships among Internet health information use, patient behavior and self efficacy in newly diagnosed cancer patients who contact the National Cancer Institute's NCI Atlantic Region Cancer Information Service (CIS). Proc AMIA Symp.

[54] Ouschan R, Sweeney JC, Johnson LW (2000). Dimensions of patient empowerment: implications for professional services marketing. Health Mark Q.

[55] Hain DJ, Sandy D (2013). Partners in care: patient empowerment through shared decision-making. Nephrol Nurs J.

[56] Williams T (2002). Patient empowerment and ethical decision making: the patient/partner and the right to act. Dimens Crit Care Nurs.

[57] Clarke E, Puschner B, Jordan H, Williams P, Konrad J, Kawohl W, Bär A, Rössler W, Del Vecchio V, Sampogna G, Nagy M, Süveges A, Krogsgaard Bording M, Slade M (2015). Empowerment and satisfaction in a multinational study of routine clinical practice. Acta Psychiatr Scand.

[58] Areni CS, Lutz RJ (1988). The role of argument quality in the elaboration likelihood model. Advances in Consumer Research.

[59] Chaiken S, Eagly AH (1976). Communication modality as a determinant of message persuasiveness and message comprehensibility. Journal of Personality and Social Psychology.

[60] Lin T, Hwang L, Lai Y (2017). Effects of argument quality, source credibility and self-reported diabetes knowledge on message attitudes: an experiment using diabetes related messages. Health Info Libr J.

[61] Raghunathan S (1999). Impact of information quality and decision-maker quality on decision quality: a theoretical model and simulation analysis. Decision Support Systems.

[62] Chen C, C.S. Ku E (2013). Bridging indistinct relationships and online loyalty: evidence from online interest-based communities. Online Information Review.

[63] Tormala ZL, Briñol P, Petty RE (2006). When credibility attacks: The reverse impact of source credibility on persuasion. Journal of Experimental Social Psychology.

[64] Zhu DH, Chang YP, Luo JJ (2016). Understanding the influence of C2C communication on purchase decision in online communities from a perspective of information adoption model. Telematics and Informatics.

[65] Syn SY, Kim SU (2014). The impact of source credibility on young adults' Health information activities on facebook: Preliminary findings. Proc. Am. Soc. Info. Sci. Tech.

[66] Xiao N, Sharman R, Rao H, Upadhyaya S (2014). Factors influencing online health information search: An empirical analysis of a national cancer-related survey. Decision Support Systems.

[67] Ghaddar SF, Valerio MA, Garcia CM, Hansen L (2012). Adolescent health literacy: the importance of credible sources for online health information. J Sch Health.

[68] Bates BR, Romina S, Ahmed R, Hopson D (2006). The effect of source credibility on consumers' perceptions of the quality of health information on the Internet. Med Inform Internet Med.

[69] Lin CA, Atkin DJ, Cappotto C, Davis C, Dean J, Eisenbaum J, House K, Lange R, Merceron A, Metzger J, Mitchum A, Nicholls H, Vidican S (2015). Ethnicity, digital divides and uses of the Internet for health information. Computers in Human Behavior.

[70] Pant S, Deshmukh A, Murugiah K, Kumar G, Sachdeva R, Mehta JL (2012). Assessing the credibility of the "YouTube approach" to health information on acute myocardial infarction. Clin Cardiol.

[71] Wei M (2013). The associations between health literacy, reasons for seeking health information, and information sources utilized by Taiwanese adults. Health Education Journal.

[72] Tennant B, Stellefson M, Dodd V, Chaney B, Chaney D, Paige S, Alber J (2015). eHealth literacy and Web 2.0 health information seeking behaviors among baby boomers and older adults. J Med Internet Res.

[73] Slim K, Bazin J (2019). From informed consent to shared decision-making in surgery. J Visc Surg.

[74] Chang H, Li F, Lin C (2019). Factors Influencing Implementation Of Shared Medical Decision Making In Patients With Cancer. PPA.

[75] Seo J, Goodman MS, Politi M, Blanchard M, Kaphingst KA (2016). Effect of Health Literacy on Decision-Making Preferences among Medically Underserved Patients. Med Decis Making.

[76] Hur K, Kim TT, Karatepe OM, Lee G (2017). An exploration of the factors influencing social media continuance usage and information sharing intentions among Korean travellers. Tourism Management.

[77] Sudbury-Riley L, FitzPatrick M, Schulz PJ (2017). Exploring the Measurement Properties of the eHealth Literacy Scale (eHEALS) Among Baby Boomers: A Multinational Test of Measurement Invariance. J Med Internet Res.

[78] Foley H, Steel A (2017). Patient perceptions of patient-centred care, empathy and empowerment in complementary medicine clinical practice: A cross-sectional study. Advances in Integrative Medicine.

[79] Krause N, Riemann-Lorenz K, Steffen T, Rahn AC, Pöttgen J, Stellmann J, Köpke S, Friede T, Icks A, Vomhof M, Temmes H, van de Loo M, Gold SM, Heesen C (2021). Study protocol for a randomised controlled trial of a web-based behavioural lifestyle programme for emPOWERment in early Multiple Sclerosis (POWER@MS1). BMJ Open.

[80] Rahn AC, Wenzel L, Icks A, Stahmann A, Scheiderbauer J, Grentzenberg K, Vomhof M, Montalbo J, Friede T, Heesen C, Köpke Sascha (2021). Development and evaluation of an interactive web-based decision-making programme on relapse management for people with multiple sclerosis (POWER@MS2)-study protocol for a randomised controlled trial. Trials.

[81] Changsha Ranxing Information Technology Co., Ltd.

[82] Hair Jr. JF, Matthews LM, Matthews RL, Sarstedt M (2017). PLS-SEM or CB-SEM: updated guidelines on which method to use. IJMDA.

[83] Ringle, Sarstedt, Straub (2012). Editor's Comments: A Critical Look at the Use of PLS-SEM in "MIS Quarterly". MIS Quarterly.

[84] Hair JF, Sarstedt M, Ringle CM, Mena JA (2011). An assessment of the use of partial least squares structural equation modeling in marketing research. J Acad Mark Sci.

[85] Fornell C, Larcker Df (1981). Evaluating Structural Equation Models with Unobservable Variables and Measurement Error. Journal of Marketing Research.

[86] Bagozzi RP, Yi Y (1988). On the evaluation of structural equation models. JAMS.

[87] Podsakoff PM, Organ DW (1986). Self-Reports in Organizational Research: Problems and Prospects. Journal of Management.

[88] Liang, Saraf, Hu, Xue (2007). Assimilation of Enterprise Systems: The Effect of Institutional Pressures and the Mediating Role of Top Management. MIS Quarterly.

[89] Keil M, Tan BCY, Wei K, Saarinen T, Tuunainen V, Wassenaar A (2000). A Cross-Cultural Study on Escalation of Commitment Behavior in Software Projects. MIS Quarterly.

[90] Zhang X, Ma L, Xu B, Xu F (2019). How social media usage affects employees’ job satisfaction and turnover intention: An empirical study in China. Information & Management.

[91] Hwang Y (2010). The moderating effects of gender on e-commerce systems adoption factors: An empirical investigation. Computers in Human Behavior.

[92] Zhao X, Lynch JG, Chen Q (2010). Reconsidering Baron and Kenny: Myths and Truths about Mediation Analysis. J Consum Res.

[93] MacKinnon DP, Lockwood CM, Hoffman JM, West SG, Sheets V (2002). A comparison of methods to test mediation and other intervening variable effects. Psychol Methods.

[94] Shin KS, Lee E (2018). Relationships of health literacy to self-care behaviors in people with diabetes aged 60 and above: Empowerment as a mediator. J Adv Nurs.

[95] Crondahl K, Eklund Karlsson L (2016). The Nexus Between Health Literacy and Empowerment. SAGE Open.

[96] Finbråten Hanne Søberg, Nordström Gun, Pettersen KS, Trollvik A, Wilde-Larsson B, Guttersrud (2020). Explaining variance in health literacy among people with type 2 diabetes: the association between health literacy and health behaviour and empowerment. BMC Public Health.

