# Supplementary material for: Understanding Health Empowerment From the Perspective of Information Processing: Questionnaire Study
Source: J Med Internet Res. 2022 Jan 11;24(1):e27178. doi: 10.2196/27178 (PMC8790685; doi:10.2196/27178)
Supplement: Multimedia Appendix 1 [file jmir_v24i1e27178_app1.doc]

**Appendix A**

Measurement Scales

| **Construct** | **Measures** | **Sources** |
| --- | --- | --- |
| Perceived Source Credibility | The provider of online health information is knowledgeable. | (Hur et al., 2017) |
|  | The provider is an expert on the message topic. |  |
|  | The provider of online health information is trustworthy. |  |
|  | The provider of online health information is reliable. |  |
| Perceived Argument Quality | The health information provided online is informative. | (Sussman et al., 2003) |
|  | The health information provided online is helpful. |  |
|  | The health information provided online is valuable. |  |
|  | The health information provided online is persuasive. |  |
| Perceived Informational benefits | By searching for online health information, I feel better informed as a patient. | (van Uden-Kraan et al., 2009) |
|  | By searching for online health information, I understand my illness better. |  |
|  | By searching for online health information, I have a clearer picture about my illness. |  |
|  | By searching for online health information, I feel like I have more (correct) knowledge at my disposal to deal better with my illness. |  |
| Perceived decision-making benefits | Online health information is helpful to decide what questions to ask during doctor appointments. | (Seçkin, 2010) |
|  | Online health information is helpful to decide on treatment choices and make decisions. |  |
|  | Online health information is helpful to decide whether to question or challenge doctors’ decisions. |  |
|  | Online health information is helpful to decide whether to obtain second opinion from another doctor. |  |
| Health empowerment | I feel more in control of my health. | (Bann et al., 2010) |
|  | I know what to do to take care of my health problem. |  |
|  | I believe that my health problem will improve. |  |
|  | I advocate more for myself. |  |
|  | I have techniques that I can use when my symptoms get worse. |  |
| Health literacy | I know what health resources and information are available on the Internet. | (Sudbury-Riley et al., 2017) |
|  | I know where to find helpful health resources and information on the Internet. |  |
|  | I know how to find helpful health resources and information on the Internet. |  |
|  | I know how to use the Internet to answer my questions about health. |  |
|  | I know how to use the health information I find on the Internet to help me. |  |
|  | I have the skills I need to evaluate the health resources and information I find on the Internet. |  |
|  | I can tell high-quality health resources and information from low-quality health resources and information on the Internet. |  |
|  | I feel confident in using information from the Internet to make health decisions. |  |

Bann, C. M., Sirois, F. M., & Walsh, E. G. (2010). Provider support in complementary and alternative medicine: exploring the role of patient empowerment. *Journal of Alternative & Complementary Medicine, 16*(7), 745-752. doi: 10.1089/acm.2009.0381

Hur, K., Kim, T. T., Karatepe, O. M., & Lee, G. (2017). An exploration of the factors influencing social media continuance usage and information sharing intentions among Korean travelers. *Tourism Management, 63*, 170-178. doi: 10.1016/j.tourman.2017.06.013

Seçkin, G. (2010). Cyber patients surfing the medical web: Computer-mediated medical knowledge and perceived benefits. *Computers in Human Behavior, 26*(6), 1694-1700. doi: 10.1016/j.chb.2010.06.018

Sudbury-Riley, L., FitzPatrick, M., & Schulz, P. J. (2017). Exploring the Measurement Properties of the eHealth Literacy Scale (eHEALS) Among Baby Boomers: A Multinational Test of Measurement Invariance. *Journal of Medical Internet Research, 19*(2), e53. doi: 10.2196/jmir.5998

Sussman, S. W., & Siegal, W. S. (2003). Informational influence in organizations: An integrated approach to knowledge adoption. *Information Systems Research, 14*(1), 47-65. doi: 10.1287/isre.14.1.47.14767

van Uden-Kraan, C., Drossaert, C., Taal, E., Seydel, E. R., & van de Laar, M. A. F. J. (2009). Participation in online patient support groups endorses patients' empowerment. *Patient Education and Counseling, 74*(1), 61–69. doi: 10.1016/j.pec.2008.07.044
