# Supplementary material for: Understanding Health Empowerment From the Perspective of Information Processing: Questionnaire Study
Source: J Med Internet Res. 2022 Jan 11;24(1):e27178. doi: 10.2196/27178 (PMC8790685; doi:10.2196/27178)
Supplement: Multimedia Appendix 2 [file jmir_v24i1e27178_app2.doc]

**Common Method Bias Analysis**

| **Construct** | **Indicator** | **Substantive Factor loadings (R1)** | **R1^2^** | **Method Factor Loading (R2)** | **R2^2^** |
| --- | --- | --- | --- | --- | --- |
| AQ | AQ1 | 0.689 | 0.475 | 0.070 | 0.005 |
|  | AQ2 | 0.887 | 0.787 | -0.083 | 0.007 |
|  | AQ3 | 0.853 | 0.728 | -0.076 | 0.006 |
|  | AQ4 | 0.615 | 0.378 | 0.109 | 0.012 |
| SC | SC1 | 0.767 | 0.588 | 0.017 | 0.000 |
|  | SC2 | 0.705 | 0.497 | 0.068 | 0.005 |
|  | SC3 | 0.864 | 0.746 | -0.068 | 0.005 |
|  | SC4 | 0.829 | 0.687 | -0.014 | 0.000 |
| PIB | PIB1 | 0.668 | 0.446 | 0.095 | 0.009 |
|  | PIB2 | 0.788 | 0.621 | 0.086 | 0.007 |
|  | PIB3 | 0.888 | 0.789 | -0.086 | 0.007 |
|  | PIB4 | 0.700 | 0.490 | -0.098 | 0.010 |
| PDB | PDB1 | 0.835 | 0.697 | -0.155 | 0.024 |
|  | PDB2 | 0.626 | 0.392 | 0.151 | 0.023 |
|  | PDB3 | 0.741 | 0.549 | -0.023 | 0.001 |
|  | PDB4 | 0.749 | 0.561 | 0.015 | 0.000 |
| EM | EM1 | 0.783 | 0.613 | -0.062 | 0.004 |
|  | EM2 | 0.737 | 0.543 | 0.037 | 0.001 |
|  | EM3 | 0.735 | 0.540 | -0.029 | 0.001 |
|  | EM4 | 0.726 | 0.527 | 0.025 | 0.001 |
|  | EM5 | 0.690 | 0.476 | 0.028 | 0.001 |
| HL | HL1 | 0.836 | 0.699 | -0.069 | 0.005 |
|  | HL2 | 0.850 | 0.723 | -0.024 | 0.001 |
|  | HL3 | 0.774 | 0.599 | 0.014 | 0.000 |
|  | HL4 | 0.682 | 0.465 | 0.031 | 0.001 |
|  | HL5 | 0.587 | 0.345 | 0.144 | 0.021 |
|  | HL6 | 0.794 | 0.630 | -0.041 | 0.002 |
|  | HL7 | 0.831 | 0.691 | -0.094 | 0.009 |
|  | HL8 | 0.706 | 0.498 | 0.059 | 0.003 |
| Average | |  | 0.579 |  | 0.006 |
